# Editorial: Emotional Function of Sociability in Fish

**DOI:** 10.3389/fnbeh.2022.909352

**Published:** 2022-05-04

**Authors:** Marta C. Soares, Caio Maximino

**Affiliations:** ^1^CIBIO, Centro De Investigação em Biodiversidade e Recursos Genéticos, InBIO Laboratório Associado, Campus de Vairão, Universidade Do Porto, Vairão, Portugal; ^2^BIOPOLIS Program in Genomics, Biodiversity and Land Planning, CIBIO, Campus de Vairão, Vairão, Portugal; ^3^Laboratório de Neurociências e Comportamento “Frederico Guilherme Graeff”, Faculdade De Psicologia, Instituto De Estudos em Saúde e Biológicas, Universidade Federal Do Sul e Sudeste Do Pará, Marabá, Brazil; ^4^Grupo De Pesquisas em Neurociências, Comportamento & Cognição, Universidade Federal Do Sul e Sudeste Do Pará, Marabá, Brazil

**Keywords:** sociabilities, teleost fish, emotionality, social plasticity, stress

This Research Topic aimed to gather different contributions regarding the crucial role that social ‘and emotional function plays in the neurochemical and neurophysiological organization of social and emotion-like behavior in fish.

In aiming to contribute to a substantial increase of knowledge regarding fish abilities to engage in complex social behavior, Silveira et al. investigated the memory retention related to agonistic interactions in the dusky damselfish. The authors explore the relevance of familiarity on memory retention duration and hypothesize that fish can remember familiar conspecifics and hence behave less aggressively. Evidence demonstrated that these fish retained visual memory of a familiar opponent for at least 5 days, but in other contexts (appetitive and aversive place conditioning) retention went beyond 15 days, which confirmed their ability to recognize, memorize, and change their behavioral response selectively in future encounters.

Because interactions between individuals may vary in aggressiveness or stress, the outcome contributes to elicit an emotional state: for instance, the outcome of an agonist encounter is either becoming a winner or a loser (Oliveira et al., 2009), and, at the long term these encounters can lead to the formation of dominance hierarchies. Hierarchies, through social plasticity, can induce changes in systems related to emotionality (Maruska et al., 2019). Bozi et al. aimed to test if status in a dominance-subordinate hierarchy can increase anxiety-like behavior in the zebrafish and discovered that not only both male and female zebrafish could establish dominance, but also both subordinate males and females seem to display increased anxiety-like behavior in the novel tank test after the establishment of the hierarchy.

In humans and other animals, individual emotional states can elicit similar behavioral responses to other group members, thus being characterized as contagious (Hatfield et al., 2011). Unsurprisingly, emotional contagion has been traditionally considered to be a prerogative of mammals and birds (for example Palagi et al., 2020) but Burbano Lombana et al. provide an emergent empirical contribution that adds to the few recent findings demonstrating otherwise. Here the authors investigate the role of emotional contagion in zebrafish by testing if individual emotional states can be transmitted to group members, by pharmacologically modulating anxiety-related behaviors of a single fish through citalopram administration and then observing if the altered behavior would spread to a group of untreated conspecifics.The authors found that by reducing anxiety-like behavior in the treated individual (in the form of reduced geotaxis), this behavioral pattern was rapidly generalized to the remaining untreated conspecifics. Not only this study demonstrates that emotional contagion is a feature found in zebrafish, but it goes beyond in showing that a single introduction of a stress/anxiety reduction factor is enough to produce a collective response in fish.

The final article in the collection, by Ogawa and Parhar, reviews the role of a putative hub in the emotion-sociality interface, the habenula, in fish behavior, discussing the literature on the neuroanatomical organization of the habenula in mammals and fish, its behavioral functions, and convincingly arguing that this structure should be considered an interface between social behavior network and the mesolimbic reward system. Moreover, given the role of the habenula in fear- and anxiety-like behavior, this structure is likely to also produce an interface with brain systems that are associated with defense (do Carmo Silva et al., 2018), thus representing an important cynosure that links stress, emotionality, and sociality (see Soares et al., 2018). Ogawa and Parhar also refers to the habenula as the target for the modulation of different neuropeptide and other neuroendocrine systems that are involved in the complex interactions between emotionality and sociality. Thus, the habenula and its projections to and from other structures in social, reward, and defensive systems are likely to play a crucial role in the social plasticity of emotion-like behavior in fish, as well as the effects of stress on sociality.

Overall, the articles in this Topic underline both the conservation of complex social and emotional behavior in fish, and the uniqueness that fish species can lend to research in the field of social and affective neuroscience. The articles present novel and exciting avenues for social plasticity research - and, given the rapidly expanding use of zebrafish and other fish species as model organisms in neuroscience, offer very interesting endpoints to expand neurobiological research.

## Author Contributions

All authors listed have made a substantial, direct, and intellectual contribution to the work and approved it for publication.

## Funding

MS is supported by Portuguese National Funds through Fundação para a Ciência e Tecnologia (DL57/2016/CP1440/CT0019). CM is the recipient of a Conselho Nacional de Desenvolvimento Científico e Tecnológico (CNPq/Brazil) productivity grant (#302998/2019-5).

## Conflict of Interest

The authors declare that the research was conducted in the absence of any commercial or financial relationships that could be construed as a potential conflict of interest.

## Publisher's Note

All claims expressed in this article are solely those of the authors and do not necessarily represent those of their affiliated organizations, or those of the publisher, the editors and the reviewers. Any product that may be evaluated in this article, or claim that may be made by its manufacturer, is not guaranteed or endorsed by the publisher.
